# Application of an improved Discrete Salp Swarm Algorithm to the wireless rechargeable sensor network problem

**DOI:** 10.3389/fbioe.2022.923798

**Published:** 2022-09-20

**Authors:** Zhang Yi, Zhou Yangkun, Yu Hongda, Wang Hong

**Affiliations:** ^1^ College of Electrical and Computer Science, Jilin Jianzhu University, Changchun, China; ^2^ Information Center of the Ministry of Natural Resources, Beijing, China

**Keywords:** wireless rechargeable sensor network, swarm intelligence, salp swarm algorithm, ant colony system, optimization

## Abstract

This paper presents an improved Discrete Salp Swarm Algorithm based on the Ant Colony System (DSSACS). Firstly, we use the Ant Colony System (ACS) to optimize the initialization of the salp colony and discretize the algorithm, then use the crossover operator and mutation operator to simulate the foraging behavior of the followers in the salp colony. We tested DSSACS with several algorithms on the TSP dataset. For TSP files of different sizes, the error of DSSACS is generally between 0.78% and 2.95%, while other algorithms are generally higher than 2.03%, or even 6.43%. The experiments show that our algorithm has a faster convergence speed, better positive feedback mechanism, and higher accuracy. We also apply the new algorithm for the Wireless rechargeable sensor network (WRSN) problem. For the selection of the optimal path, the path selected by DSSACS is always about 20% shorter than the path selected by ACS. Results show that DSSACS has obvious advantages over other algorithms in MCV’s multi-path planning and saves more time and economic cost than other swarm intelligence algorithms in the wireless rechargeable sensor network.

## 1 Introduction

In recent years, meta-heuristic techniques have solved many problems with the rapid development of meta-heuristic algorithms (Zhang et al., 2018). There are two main reasons why meta-heuristic algorithms can be competitive with practical problems such as single-objective and multi-objective optimization problems (Cui et al., 2021a). Firstly, people used mathematical methods to solve practical problems before the proposed meta-heuristic optimization technology. However, practical issues are usually continuous or discrete. Some issues may also have certain constraints (Abbassi et al., 2019). Secondly, a new method is urgently being created because the determinism of traditional mathematical methods often leads to inefficiencies in solving practical problems, then the metaheuristic algorithm was invented which shows advantages of flexibility and universality faced with many large-scale multi-modal, discontinuous, and non-differentiable issues in the real world, thus can avoid falling into local optimum and can be widely used and applied to various scientific problems (Mirjalili and Lewis, 2016).

Meta-heuristic algorithms can be divided into two categories. One is the evolutionary algorithm such as the Genetic Algorithm (GA) (Chatterjee et al., 1996), one of many people’s most fundamental algorithms and is considered an evolutionary algorithm. GA uses the newly generated population to replace the old population to accomplish the evolution of the population. The evolutionary algorithm also includes Memetic Algorithm (MA) (Pablo, 1989), Multi-Objective Evolutionary Algorithm (MOEA) (Deb et al., 2002), etc. An evolutionary algorithm is a mature global optimization (Nedjah et al., 2021) method with high robustness and is widely applicable, which has the characteristics of self-organization, self-adaptation, and self-learning. It can effectively deal with complex problems that are difficult to be solved by traditional optimization algorithms (such as NP-hard optimization problems (Zhang et al., 2022)) without being limited by the characteristics of the issues. The other is the swarm intelligence algorithm. Scientists have studied the group behavior of organisms in nature by using bionic technology to simulate the social behavior of biological populations. As a result, they found that the simulated algorithm can solve practical problems, such as the Ant colony algorithm (Ant Colony Optimization, ACO) (Dorigo and Di Caro, 1999), which is created by studying the cooperative foraging behavior of ants, and Cuckoo search (CS), a Swarm intelligence algorithm that can solve multi-objective optimization problems (Cui et al., 2019a).

The swarm intelligence algorithm is simple to be implemented with no centralized control constraints (Cui et al., 2019b). And it will not be affected by individual failures, which can influence the solution of the entire problem. Swarm intelligence algorithms are generally used for two purposes. One is to solve persistent problems, and the other is to solve discrete problems. The swarm intelligence algorithms that are used to solve continuous problems include Artificial Bee Colony (ABC) (Karaboga and Basturk, 2008), Whale Optimization Algorithm (WOA) (Mafarja and Mirjalili, 2017), Grey Wolf Optimizer (GWO) (Mirjalili et al., 2014; Sivaramakrishnan et al., 2020), Salp Swarm Algorithm (SSA) (Mirjalili et al., 2017), etc. These algorithms are generally used for the optimization of specific functions. The standard swarm intelligence algorithms for solving discrete problems include the ant colony algorithm (ACO) and discrete particle swarm algorithm (PSO). These algorithms are used to solve combinatorial optimization problems such as TSP (Traveling Salesman Problem) and vehicle routing problems (VRP).

Swarm intelligence algorithms have excellent applicability and plasticity. The improved swarm intelligence algorithm will perform better. For example, if quantum computing is introduced into the monarch butterfly optimization (MBO), the monarch butterfly can find a shorter path (Yi et al., 2020). Scientists have discovered that many swarm intelligence algorithms can solve persistent problems and have the potential to solve discrete problems. Particle Swarm Optimization (PSO) (Eberhart and Kennedy, 1995) is an algorithm created by imitating the social behavior of geese, and it has been proved its excellent role in the field of continuous problems for a long time ago. Its excellent applicability is often improved in other fields such as medicine (Zemmal et al., 2021). TSP (Traveling Salesman Problem) is a classic combinatorial optimization problem with the characteristic of NP-hard and discrete (Bellman, 1962; Bellmore and Nemhauser, 1968). In optimizing the outlier scores,Sharon Femi and Ganesh Vaidyanathan. (2022) used chicken swarm optimization (CSO) to increase the deviation between the outliers and inliers according to the chicken competition. Wang et al. (2003) designed a discrete particle swarm optimization algorithm with a faster convergence speed using exchange operators and exchange sequences (Shi et al., 2007). Fei et al. (2014) improved the Artificial Fish Swarm Algorithm (AFSA) created by Li, which was used to research TSP problems in faster early convergence speed and quicker local optimum found.

The research in this paper focuses on discretization improvement and optimization of the salp swarm algorithm (SSA). SSA is a new swarm intelligence algorithm proposed by Mirjalili et al. (2017), based on the swarm foraging behavior of salps in the ocean. tested their algorithm in single-objective and multi-objective optimization problems, and the salp swarm algorithm showed high convergence and strong searchability. More importantly, for high-dimensional data, SSA also outperforms (Cui et al., 2021b). In addition, it is also suitable for the application of wings and ship propellers. At present, SSA is applied to various cases and problems, such as workshop scheduling problems (Liu et al., 2022), wireless sensor network (WSN) positioning problems (Kanoosh et al., 2019), grid distributed power optimization problems (Pantoja and Quijano, 2011), and multi-level threshold Image segmentation problems (Abualigah et al., 2022) et al. Our experiments show that the unique chain structure of SSA has a positive effect on improving the convergence speed and accuracy of the algorithm, and SSA is easy to be transformed, and the transformed algorithm has great advantages in solving discrete problems.

Moreover, SSA has excellent advantages in optimizing single, multi-modal, and composite benchmark functions. However, the salp swarm algorithm also has shortcomings (Faris et al., 2018). The salp swarm algorithm has low search accuracy and slow convergence speed and quickly falls into the local optimum. Because the initial population of the salp swarm algorithm is randomly formed, there is a lack of correlation between the populations and the overall lack of purpose. Therefore, the convergence speed and stability in the early search stage are not brilliant.

This paper proposes an improved salp swarm discrete algorithm based on the ant colony system (DSSACS). Firstly, the crossover and mutation operators are introduced to make SSA suitable for discrete problems. The pheromone matrix is used to initialize the salp population to make the leaders more purposeful and improve the relevance of leaders and followers. The ant colony system is so mature that many algorithms reference it (Deng et al., 2020; Zhang et al., 2020). Moreover, it can promote early search efficiency. DSSACS has a faster convergence speed and higher solution accuracy compared with ACO. The new algorithm is suitable for discrete problems, and its efficiency is improved significantly. We apply the improved algorithm in TSP problems and get an efficient result. (DSSACS is superior to other swarm intelligence algorithms in terms of stability and convergence speed. In TSP datasets with different numbers of cities, DSSACS has better performance than other algorithms).

However, the effectiveness of an algorithm in solving a set of problems does not guarantee its success in a different stage of the issue. Consequently, we apply the improved algorithm in wireless rechargeable sensor networks. The point of wireless rechargeable sensor networks (Fu et al., 2016) is a new scientific research topic. It originated from a technology proposed by Kurs et al. (2007). The main content is magnetic resonance coupling technology to achieve remote contactless charging, wireless charging. It is necessary to have a wireless charging device to charge the wireless sensor remotely (Cheon et al., 2011) in order to make the wireless sensor network running permanently or have a longer life cycle. Xie et al. (2012) found that if a wireless charging vehicle (WCV) is used to assess the wireless sensor network, after a while, the wireless rechargeable sensor network will form a dynamic balance, which keeps the WRSN in the running state forever. Research on this goes far beyond that. Scientists began to study the mathematical model of WRSN in the case of multiple charging vehicles or mobile chargers, constrain the capacity or the rate of MCs (Dai et al., 2014; Shu et al., 2016), plan the paths of various MCs, and obtain a scheme with the minimum moving distance of multiple MCs (Xu et al., 2014). Swarm intelligence algorithms also have made extraordinary contributions in this field. The improved firefly algorithm (IFA) is a swarm intelligence algorithm. Sun et al. (2017), Yang et al. (2018) used IFA to deploy the wireless charging nodes (WCNs) of the WRSN reasonably, then made the efficiency and the Coverage of is maximized at the same time. (Chen and Jiang, 2016) applied the particle swarm algorithm (PSO) to the deployment of chargers in WRSN. They optimized the swarm intelligence into the charger’s location and even the direction of the antenna. Lyu et al. (2019) proposed a hybrid particle swarm optimization genetic algorithm (HPSOGA), and they used the limited energy of the charging device as a constraint. The improved swarm intelligence algorithm was used to ensure the periodic change of charging. Feng et al. (2020) used newborn particle swarm optimization (NPSO) to add nascent particles to the swarm to optimize the charging scheduling of WRSN. Finally, they found that NPSO improved energy utilization and reduced the mortality of nodes. It can be seen that the swarm intelligence algorithm plays an essential role in the study of the WRSN problem. However, few people apply the swarm intelligence algorithm to MCV charging path planning in WRSN. Generally speaking, good charging path planning can primarily reduce the cost of time and money. Therefore, this paper studied a WRSN mathematical model with multiple traveling salesman problems (MTSP) in the case of numerous MC/MCV and used DSSACS for optimization.

The structure of this paper is as follows: In Section 2, the basic concepts of the TSP problem, MTSP problem, and WRSN problem are described, the WRSN model is established, and the problems and calculation formula we need to solve are described. In Section 3, we describe the idea of improving the salp swarm algorithm (SSA), propose a new algorithm——the ant-colony-system-based salp discrete optimization algorithm, and explain the parameter selection for our algorithm to solve NP-hard problems. In Section 4, we conducted experiments. Firstly, we applied DSSACS to the WRSN problem to obtain the results and compared it with other algorithms. Then we reached the DSSACS algorithm with different algorithms on multiple TSP problems. Section V is the conclusion of our work.

## 2 Problem description

We established the mathematical model of WRSN in 2.1, introduced the concept of MTSP in section 2.2.

### 2.1 Wireless rechargeable sensor network model

**FIGURE 1:**
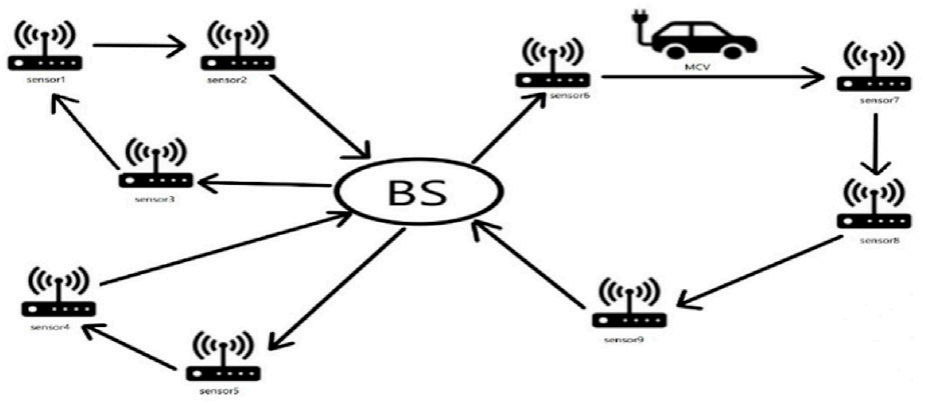
The Schematic diagram of WRSN.

WRSN model is a mathematical model based on the two-dimensional plane. WRSN consists of a fixed transmission station (BS), wireless rechargeable sensors, and *n* mobile charging vehicles (MCVS). The base station does not move and exchanges data with wireless sensors—the base station is typically located in the general center of a wireless rechargeable network. If one of the wireless sensors fails or the power level falls below the sensor’s minimum threshold, the entire WRSN will fail. Wireless sensors need to be periodically charged using a mobile charger (MC) (Liang et al., 2014) to make WRSN permanent. The most common MC is MCV. MCV starts from BS and successfully captures multiple wireless sensor nodes in a charging cycle. After completing the charging task, MCV returns to BS for its charging. MCVs will not interfere with each other during their own charge cycle. Multiple MCVS working together on charging commissions can turn the entire process into an MTSP problem. The WRSN charging problem can be represented in Figure 1. The MTSP is meaningless if there are no constraints for multi-MCV charging WRSN networks. Moreover, only the WRSN model with constraints can approach the problem (Pan and Wang, 2006). This paper studies specific power consumption rates and the total battery capacity of the wireless sensors in WRSN. Each MCV is assigned charging tasks as evenly as possible to calculate the absolute minimum operating distance after completing a cycle task to minimize the maximum battery capacity of the sensor in the network.

Suppose all wireless sensor nodes $S_{1}、S_{2}、S_{3}\cdot \cdot \cdot S_{n}$ . If an MCV is used to charge all wireless sensor nodes, then a charging cycle is defined as $BS\to S_{1}\to S_{2}\to S_{3}\to \cdot \cdot \cdot \to S_{n}\to BS$ , equivalent to a TSP problem of *n*+1 nodes. However, there is continuous energy consumption, and the energy consumption and minimum capacity of wireless sensors need to be calculated in the mathematical model of WRSN. The multi-mcv charging problem can be regarded as an extension of the single MCV charging problem. Each MCV not only needs to complete its charging cycle but also needs to satisfy $\overset{m}{\underset{i}{\sum }}n_{i}=n\left(i=1,2,\ldots ,m\right)$ , where *m* represents the number of MCV and $n_{i}$ represents the number of sensor nodes required to charge the *i*th MCV.

We first deal with the case that a single MCV charges WRSN. It can be simply seen as a TSP problem. At a given speed, the charging cycle and the running distance of MCV have the following relationship:

$$t_{Ni}=\frac{D_{Ni}}{R-U_{i}}$$ (1.1)

$$D_{Ni}=U_{i}\text{*}\left(T_{0}+\overset{n}{\underset{j=n+1}{\sum }}t_{N-1}+\overset{i-1}{\underset{j=0}{\sum }}t_{N,j}\right).i=1,2,3,\cdots ,n-1$$ (1.2)

$$t_{Ni}=\frac{D_{Nn}}{R-U_{n}}$$ (1.3)

$$D_{Nn}=U_{i}\text{*}\left(T_{0}+\overset{n-1}{\underset{j=1}{\sum }}t_{N,j}\right).i=1,2,3,\ldots ,n-1$$ (1.4)

Where $\;t_{Ni}\left(i=0,1,2,\cdot \cdot \cdot ,n\right)\;$ represents the charging time required to charge the *i*th wireless sensor in the *N*_th_ cycle, and $D_{Ni}\left(i=0,1,2,\cdot \cdot \cdot ,n\right)$ represents the electric charge required to charge the *i*th sensor in the *N*_th_ cycle. $\;U_{i}\left(i=0,1,2,\cdot \cdot \cdot ,n\right)$ is the specific power consumption rate of the *i*th sensor; *R* is the fixed charging rate of MCV; $T_{0}$ is the total time consumed by MCV when moving in the shortest path. After the *N*_th_ charging cycle, the whole WRSN system tends to be stable, and finally, the minimum bearing capacity of each sensor tends to a constant value. Set the minimum battery threshold of each sensor to *w*. If the threshold is lower than *w*, the sensor will stop working immediately. After reaching the steady-state, the battery capacity of each sensor is set as $W_{i}$ , and the MCV will dock at the base station for $t_{0}$ time after each charging cycle. Assume that when the charging car just reaches a wireless sensor, the remaining battery capacity of the wireless sensor is just equal to the minimum threshold, so there are two formulas:

$$W_{i}=w+U_{i}\cdot \left(\overset{n}{\underset{i=1}{\sum }}t_{i}-t_{i}+t_{d}\right)$$ (1.5)

$$W_{i}=w+\left(R-U_{i}\right)\cdot t_{i}$$ (1.6)

According to Eqs 1.5,1.6.

$$W_{i}=w+\frac{U_{i}\left(T_{0}+t_{0}\right)\left(R-U_{i}\right)}{R-\sum_{j=1}^{n}U_{i}}$$ (1.7)

It can be seen that when the whole WRSN system reaches the steady state, the maximum battery capacity in the sensor network can be calculated at $\underset{1\leq i\leq n}{\max}W_{i}$ .The TSP problem becomes an MTSP problem in the case of multiple MCVS. In this paper, the DSSACS algorithm is used to optimize m charging paths so that the distance of all charging paths and $\;s_{sum}=s_{1}+s_{2}+\cdot \cdot \cdot +s_{m}$ is minimum, and the maximum battery capacity of the WRSN network in this state is obtained.

### 2.2 Multi-travel salesman problem

Traveling Salesman Problem (TSP) is the most typed problem in combinatorial optimization. TSP refers to making the shortest. Total distance when the salesman starts from the starting point to sell goods in all cities and finally back to the starting point. This problem is an entirely undirected graph with weight, in which a Hamilton cycle with the lowest weight is found. Let  G=(V,E) , V={1,2, ···,i} represent the coordinates of a total of *i* cities, *E* represents the set of routes between cities, and a weight $d_{jk}\left(j,k\in i,j\neq k\right)$ expresses the distance between two cities. Obviously, a minimum Hamiltonian loop x is required, where  x∈i .

$$D_{min}=\overset{k-1}{\underset{j=1}{\sum }}d_{x_{j}x_{j+1}}+d_{x_{k}x_{1}}$$ (1.8)

To meet all kinds of specific requirements, put forward the multi-travel salesman problem (MTSP). MTSP is a particular case of TSP, is the extension and extension of TSP. The MTSP problem can be expressed as follows: *m* salesmen are going to *n* cities to sell their products. They start from the same starting point and return to the same starting point at the end of a travel cycle. The goal is to find the minimum sum of the distances traveled by all the salesmen. It can be expressed as follows:

$$Min\overset{m}{\underset{\;i=1}{\sum }}D_{min,i}$$ (1.9)

First of all, it is the combination of multiple TSP problems and requires a variety of constraints, otherwise, the results obtained are not practical. Secondly, MTSP problems have high complexity, so we use the DSSACS algorithm to solve them. Experiments show that DSSACS have significant advantages in MTSP optimization.

## 3 Algorithm improvement

In this sections, We established the principle of the salp swarm algorithm in 3.1, introduced Ant Colony System in Section 3.2, and introduced the improved algorithm DSSACS in Section 3.3.

### 3.1 The principle of the salp swarm algorithm

The Salp Swarm Algorithm is a brand algorithm proposed by Mirjalili et al. (2017). The Algorithm simulates the social behavior of salps during foraging. Mirjalili divided salps into two groups, leaders and followers. As we all know, different from other biological groups, the salps group forms a chain when foraging for food, the salps are connected end to end, and the salps in the first half of the chain, we call them leaders, they are responsible for finding food sources and guiding the salps in the back. The remaining salps are defined as followers. The followers follow closely, and each follower follows the previous follower or leader, so a chain structure is simulated. The Salp Swarm Algorithm searches in an n-dimensional search space, and each salp stores the search results in an n-dimensional vector, denoted as X .

$$X_{i}=\left[\begin{array}{c} x_{1}^{1}\;\;\;\;\;x_{2}^{1}\;\;\;\;\ldots \;\;\;\;\;x_{d}^{1}\\ x_{1}^{2}\;\;\;\;\;x_{2}^{2}\;\;\;\;\ldots \;\;\;\;\;x_{d}^{2}\\ \vdots \;\;\;\;\;\;\;\;\vdots \;\;\;\;\;\;\;\ldots \;\;\;\;\;\;\;\;\;\;\\ x_{1}^{n}\;\;\;\;\;x_{2}^{n}\;\;\;\;\ldots \;\;\;\;\;x_{d}^{n} \end{array}\right]$$

The position update formula for the leader is as follows:

$$x_{j}^{1}=\{\begin{array}{c} F_{j}+c_{1}\left(\left(ub_{j}-lb_{j}\right)c_{2}+lb_{j}\right)\;\;\;\;\;c_{3}\geq 0\\ F_{j}+c_{1}\left(\left(ub_{j}-lb_{j}\right)c_{2}+lb_{j}\right)\;\;\;\;\;\;\;\;c_{3}\leq 0\;\;\; \end{array}$$ (2.1)

Eq. 2.1 represents the position update formula of the salps leader in the j th dimension. In the formula, $x_{j}^{1}$ represents the position of the salps in the frontmost place. A leader needs to track the food source $F_{j}$ (the location coordinates of the food source), $ub_{j}$ represents the upper bound under this dimension, and $lb_{j}$ represents the lower bound. These two parameters specify the search range of the salp group. $c_{1}$ and $c_{2}$ are two random numbers in the range [0, 1] that constrain the leader’s actions to prevent getting stuck in local solutions. $c_{1}$ is a critical parameter. Its formula is as follows:

$$c_{1}=2e^{-{\left(\frac{4t}{T}\right)}^{2}}$$ (2.2)

In the formula, t represents the current number of iterations, T represents the total number of iterations, and e is a constant. It can be seen that $c_{1}$ controls the search process of the leader, focusing on exploration in the early stage of the search and more on local development in the later stage of the search.

The follower’s position update formula utilizes Newton’s kinematics formula, which is as follows:

$$x_{j}^{i}=\frac{1}{2}\left(x_{j}^{i}+x_{j}^{i-1}\right)$$ (2.3)

In the formula, i>1 , i represents the movement mode of the salps in the back, that is, moving towards the front salps. In the actual optimization of continuous problems, the location of the food source is unknown, so we use the current optimal solution to replace the food source, which solves the issue of the food source and improves the search range of SSA. The whole process of the SSA algorithm is described in algorithm.

Algorithm 1 1: initialize population and define ubj and lbj

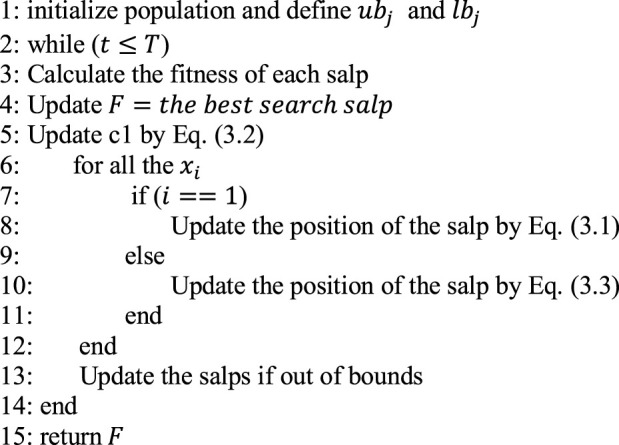

The SSA has many advantages in solving continuous problems. The SSA is a swarm intelligence algorithm that constantly develops and uses space and first has a high space utilization rate. It can effectively avoid falling into an optimal local solution, partly due to the delicate design of the coefficient $c_{1}$ . However, the search accuracy of SSA is not high, the population initialization is too random, and the initial correlation between populations is lacking, resulting in low efficiency. Moreover, using SSA to solve discrete problems is what we are eager to achieve. Can we use SSA to solve continuous problems? It is the leading research content of this paper.

### 3.2 Discrete Salp Swarm Algorithm based on the Ant Colony System

In the TSP problem, the ant colony system has a mature system. The ants are randomly assigned to the city initially, and the ants choose the next city according to the pheromone probability on the road. The more the ants’ walkthrough, the more pheromone accumulates. If there are more ants, the probability that the following ants will choose this path is higher than others. Under this positive feedback mechanism, the shortest route will be traversed by more and more ants (Yang et al., 2008). After studying the path planning of the ant colony system, it is found that ACS has some inspiration for the population initialization of SSA. The specific process of the ant colony algorithm is described below.

There are n cities, and a distance matrix D is given, where $D_{ij}$ represents the distance from the i th city to the j th city, as the weight in G=(V,E) . When ant k chooses city i , it will all travel to city j . There is a probability selection formula:

$$p_{ij}^{k}=\{\begin{array}{c} \frac{{\left[\tau_{ij}\right]}^{\alpha }\cdot {\left[\eta_{ij}\right]}^{\beta }}{\sum_{s\in allowed_{k}}{\left[\tau_{is}\right]}^{\alpha }\cdot {\left[\eta_{is}\right]}^{\beta }}\\ 0\;\;\;\;\;\;\;\;\;\;\;\;\;\;\;\;\;\;\;\;\;,otherwise \end{array}\;,\;j\in allowed_{k}$$ (2.4)

Where $\tau_{ij}$ represents the pheromone concentration between cities i and j , and correspondingly, α is used as an index to describe the importance of pheromone concentration to the ant colony. Similarly, $\eta_{ij}$ represents the visibility between cities i and j . Its value is $1/D_{ij}$ , β is used as an index to describe the importance of the distance between cities to the ant colony, and finally, $allowed_{k}$ represents the city that has not been traversed in the gather.

Obviously, an ant traveled all the cities and formed a critical path after n times probability selections. More importantly, ants will leave pheromone on the path, and pheromone also has volatilization. Then there are the following formulas:

$$\tau_{ij}\left(t+1\right)=\rho \cdot \tau_{ij}\left(t\right)+\text{Δ}\tau_{ij}$$ (2.5)

$$\text{Δ}\tau_{ij}=\overset{l}{\underset{k=1}{\sum }}\text{Δ}\tau_{ij}^{k}$$ (2.6)

$$\text{Δ}\tau_{ij}^{k}=\{\begin{array}{c} \frac{Q}{L_{k}},\;if\;ant_{k}\;traveled\;on\;edge\left(i,j\right)\\ 0,\;otherwise\;\;\;\;\;\;\;\;\;\;\;\;\;\;\;\;\;\;\;\;\;\;\;\;\;\;\;\;\;\;\;\;\;\;\;\; \end{array}$$ (2.7)

It can be seen from the formula that the concentration of pheromone left by the ants on the path is proportional to Q and inversely proportional to $L_{k}$ , where $L_{k}$ is the actual distance traveled by the ants after one traversal. The pheromone of ants volatilizes according to the volatilization coefficient ρ .

Although many algorithms can perform approximate solutions in solving NP-Hard problems, there are generally problems of low search efficiency and easy to fall into local optimal solutions (Mohan and Remya, 2014; Saenphon et al., 2019). This paper improved the salp swarm group algorithm for the first time and applied it to the resolution of discrete problems. Therefore, in the next section, we propose a novel SSA to solve the MTSP problem.

First, initialize the population of s salps, and use the leaders of the salps to imitate the ants for path selection. If the MTSP has m traveling salesman and n cities, then it is similar to that each ant starts from the starting point, passes through a qualified number of cities, and then returns to the starting point as the path of the first traveling salesman, and then starts the next time in turn. Travel until m trips are completed. Currently, each of the salps stores m segments of paths, and each path’s start and endpoints are the same. Here we use a new adaptive coefficient to define the ant colony’s pheromone heuristic factor, use the roulette wheel to choose the next city the ants go to, and finally leave the pheromone volatilization coefficient according to the length of the journey (Lloyd and Amos, 2017). Pheromones. The reason for using the ant colony system to initialize the salp group is to strengthen the correlation between the salp groups so that the salp group has a vital purpose in the early stage of the algorithm, which can not only improve the search efficiency of the algorithm but also avoid falling into a locally optimal solution. The second step is to calculate the fitness value of all salps, which is expressed as the total length of the total m-segment travel in the MTSP problem, and sort the salp population according to the calculated fitness value so that a chain structure is formed. We know that in The Salp Swarm Algorithm, the follower will have a process of moving forward to a follower in each iteration, so in our DSSACS algorithm, after sorting, the salp population is behind s/2 . Each only moves toward the salps in front of them. Here, the movement is not a concrete quantified displacement in a continuous problem but a discrete abstract motion towards the preceding salps. Here, it is necessary to encode the path stored by the salps first, cross the two encoded salps, and decode and calculate the fitness value to determine the trade-off for this move. In this way, the salps in the region with low fitness value can be optimized, and the overall convergence speed of the algorithm can be improved. Finally, we added a mutation operator suitable for MTSP to the algorithm, which improved the algorithm’s search range and development depth. Below we will explain several concepts, and parameter information will also be given in the following table.

The salp colony uses equations 2.4 to (2.7) to perform probability selection and pheromone matrix update, similar to the ant colony system. The more important thing is that the pheromone heuristic factor α is no longer a constant. We define it as follows:

$$\alpha =\varepsilon \cdot e^{\frac{t}{T}}$$ (2.8)

DSSACS focuses more on exploring the search space in the early iteration stage and not sticking to local optimization; in the later stage of the algorithm, the salps colony pays more attention to local development and mining the optimal solution.

When calculating the transition probability in 3.3.2, it is assumed that the probability of ant k choosing other cities in city i is $p\left(x_{j}\right)\left(j\neq i,j\in allowed_{k}\right)$ , and the number of untraveled cities is n . The probability of choosing j th cities is calculated like this:

$$P_{j}=\frac{p\left(x_{j}\right)}{\sum_{x=0}^{n}p\left(x_{j}\right)}$$ (2.9)

$$PP_{j}=\overset{i}{\underset{j=0}{\sum }}P_{j}\;\;\;\;\;\;\;\;\;\;\;\;\;\;\;\;\;\;\;\;\;\;\;\;i=1,\;2,\;3,\;\ldots ,\;n$$ (2.10)

The roulette will rotate n times, and a random number ψ∈ (0, 1) is generated each time. When *ψ* satisfies the following formula, city j will be selected.

$$PP_{j-1}\leq \psi <PP_{j}$$ (2.11)

Where the number of cities is n=9 , and the number of traveling salesmen is m=3 in the case. Assuming that the salp k completed an MTSP traversal, it can be encoded as:

$$X_{k}=\{\begin{array}{c} \left(0\;1\;4\;7\;0\right)\\ \left(0\;2\;5\;8\;0\right)\\ \left(0\;3\;6\;9\;0\right) \end{array}$$ (2.12)

0 represents the starting point, and the rest of the cities should satisfy $\overset{m}{\underset{i}{\sum }}n_{i}\left(i=1,2,\ldots ,m\right)$ .

If the coordinates of two cities are $D_{i}=\left(x_{i},y_{i}\right),D_{j}=\left(x_{j},y_{j}\right)$ , the distance between them is calculated by the formula:

$$D_{ij}=\sqrt[{2}]{{\left(x_{i}-x_{j}\right)}^{2}+{\left(y_{i}-y_{j}\right)}^{2}}$$ (2.13)

The calculated $D_{ij}\left(i,j=1,2,3,\cdot \cdot \cdot ,n\;∥\;i\neq j\right)$ will be stored in the matrix D. For a sequence x of length n, their overall distance:

$$Distance=\overset{n-1}{\underset{i}{\sum }}D_{x_{i},x_{i+1}}$$ (2.14)

The fitness of the path stored by a salp is calculated by Eqs 1.8, 1.9. The smaller the fitness value, the better the effect.

First, the operator will re-encode the path information stored by the followers. For this salp as represented by Eq. 2.12, its path will be re-integrated as:

$$X_{k}^{\text{∗}}=\left(\;1\;4\;7\;2\;5\;8\;3\;6\;9\;\right)$$ (2.15)

After sorting, the salps in the second half will be crossed with the previously coded salps, assuming that the two salps are A and B , as follows:

A=(1 4 7 2 5 8 3 6 9)

B=(1 5 9 3 6 7 4 2 8)

At this time, two random numbers $r_{1},r_{2}\left(r_{1}<r_{2}\right)$ are used to represent the crossed area. If the fitness of salps A is lower, then used A as a destination , and its randomly selected area does not change before and after the crossover. For example, the final generated city sequence frame is:

after=(∗ ∗ ∗ ∥2 5 8 3∥ ∗ ∗)

According to the rules of cross-transformation, B , the origination , its sequence numbers that not in $destination\left[r_{1},r_{2}\right]$ are inserted into after , and the updated after is:

after=(1 9 6 ∥2 5 8 3∥7 4 )

Every crossover will generate several new sequences, and each sequence will be decoded as (2.12), and use the fitness calculation method of 3.3.5 to compare the fitness. If the effect of the salps after moving is better than before, then this movement will be preserved.

In this paper, the mutation operator is added at the end of the algorithm, improving the search space and preventing falling into the local optimum. First, choose a salp, such as the k th salp in (2.12). For paths with m branches, each branch will use the mutation operation. When starting, randomly generate two numbers a, b(a<b) , when there are

$$Distance_{a,a+1}+Distance_{b,b+1}=Distance_{a,b}+Distance_{a+1,b+1}$$ (2.16)

Then flip x[a+1,b+1] in the single journey sequence x . The reason for this operation is to remove some intersections in the final image and improve the local exploration ability of the algorithm.

Algorithm 2 initialize population and define ubj and lbj

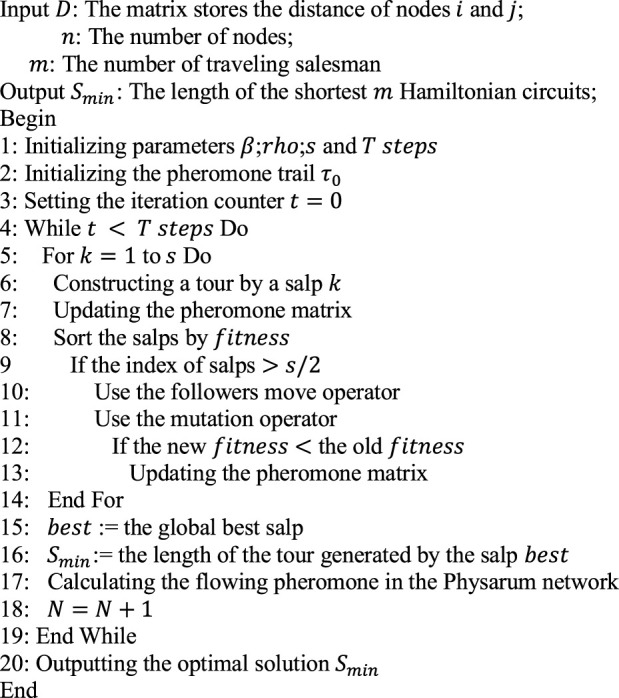

## 4 The experiment

The experiment environment is as follows: The operating system is Windows 11, CPU is AMD Ryzen 7 4700U and Inter I7-10700, memory is 16 GB. In order to test our algorithm DSSACS, we compared multiple data sets and algorithms from the TSPLIB database (HTTP://www.iwr.Uni-heidelberg.de/groups/comopt/software/TSPLIB95), and the results show that our algorithm has better performance.

We compared DSSACS with other algorithms, including genetic algorithm (GA), Ant colony algorithm (ACO), Min-Max Ant System (MMAS) (Yelmewad et al., 2019), GA-PSO-ACO (Deng et al., 2012), Tabu Search algorithm,ACO-ABC algorithm (Gündüz et al., 2015), Fast Opposite Gradient Search with Ant Colony Optimization (FOGS-ACO) algorithm (Saenphon et al., 2014), Discrete Spider Monkey Optimization (DSMO) (Akhand et al., 2020). For all TSP problems, Euclidean distance is used to quantify the effect of the algorithm. We considered the following TSP issues: Dantzig42, Att48, Eil51, Berlin52, St70, Eil76, Rat99, Rd100, etc. Part of the experimental data came from the report of Thirachit Saenphon et al. (2014), and part of the data came from the results of our operation. In terms of the gap between the final solution and the optimal value and algorithm convergence performance over time, DSSACS has obvious advantages and fast convergence speed. In addition to the performance standards of the calibration algorithm proposed in (4.4) and (4.5), a function designed based on the chi-square calibration idea -- Average deviation Rate (AVR) is also proposed in this section. Calculating the formula for $AVR=\left|\frac{S_{average}-S_{min}}{S_{min}}\right|\times 100\%$ , AVR is smaller. The algorithm has a greater probability reaches its optimal solution. For the TSP problem, our given parameters are given in Table 1.

**TABLE 1:** The parameters of the problem.

Parameter	Explanation	Value
α	The importance of the pheromone trail	Eq. 3.8
β	The importance of the heuristic information	3 for solving a MTSP (3 for solving a TSP)
rho	The pheromone evaporation rate	0.8 for solving a MTSP (0.8 for a solving TSP)
s	The number of salps	the number of cities for MTSP (TSP)
ε	The adaptive factor of α	0.5 for solving a MTSP (0.6 for solving a TSP)
fitness	The fitness of a salp	Eq. 3.14
Tsteps	The total steps of iteration	150 for solving a MTSP (300 for solving a TSP)
τ	The initial amount of pheromone in each road	N/A
D	The matrix of distance	N/A
m	The number of MCVS	4

**FIGURE 2:**
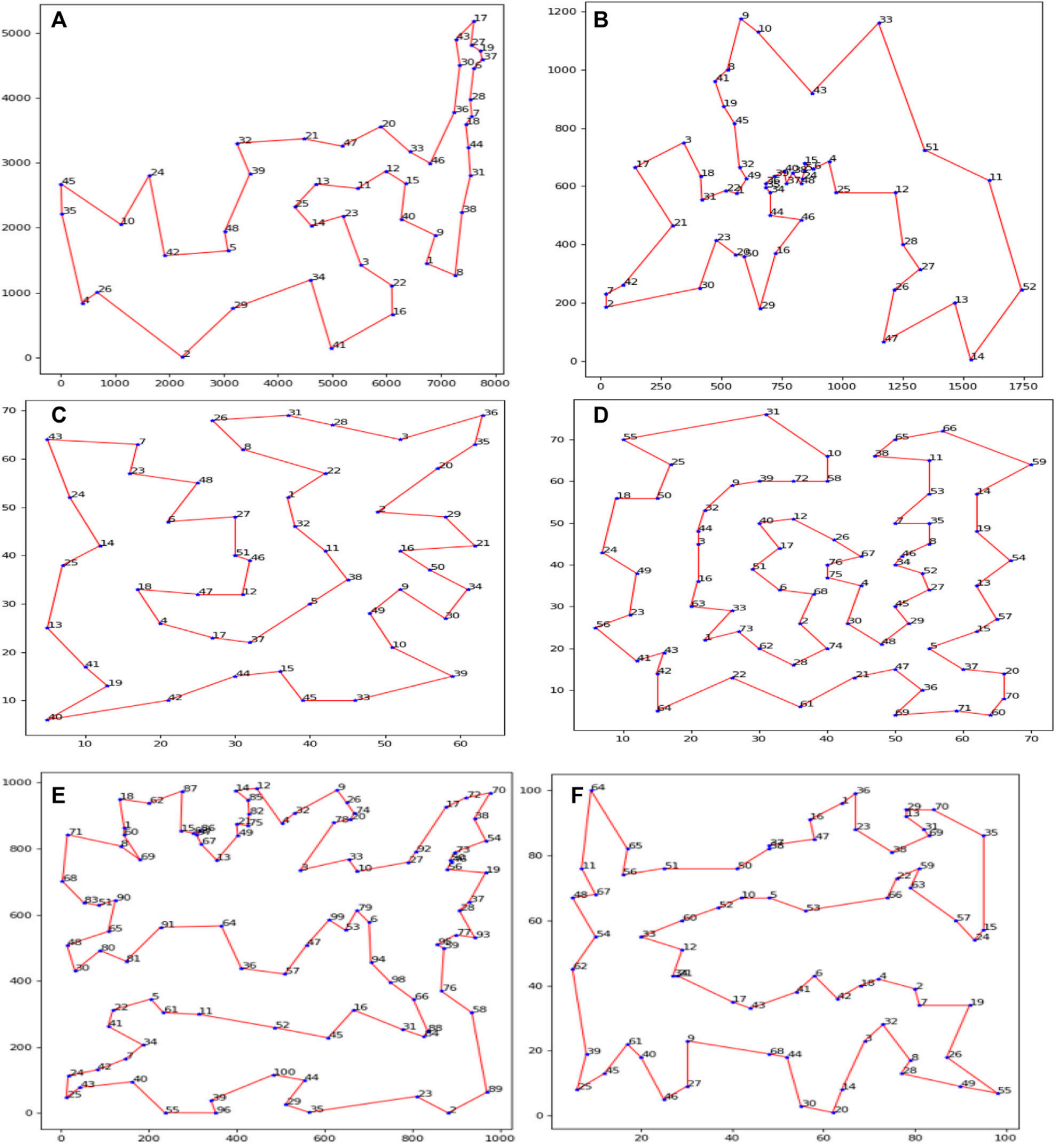
Some of the best routes generated by our algorithm **(A)** Att48, **(B)** Berlin52,**(C)** Eil51,**(D)** Eil76,**(E)** Rd100, **(F)** St70.

Firstly, our algorithm finds the optimal path for handling TSP problems, including Att48, Eil51, Berlin52, St70, Eil76, and Rd100. In Figure 2, we describe the optimal path graph searched by SDACS with enough iterations, and the serial number of cities is given in the Figure. DSSACS can find the optimal path in most TSP problems by our algorithm. Our algorithm can obtain the approximate optimal path, indicating that it has strong applicability and computing power in different TSP problems.

**FIGURE 3:**
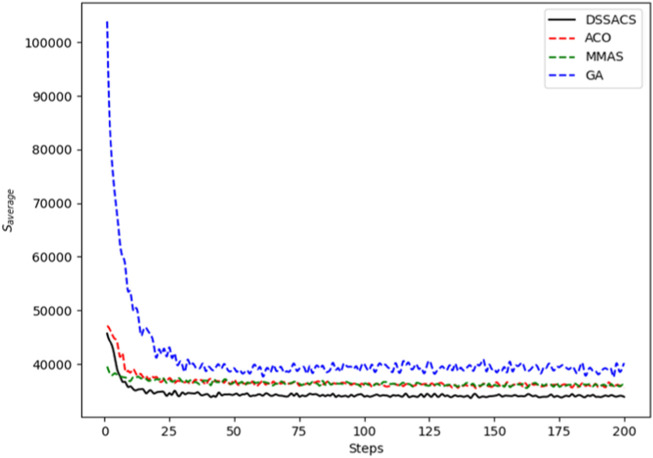
Att48.

**FIGURE 4:**
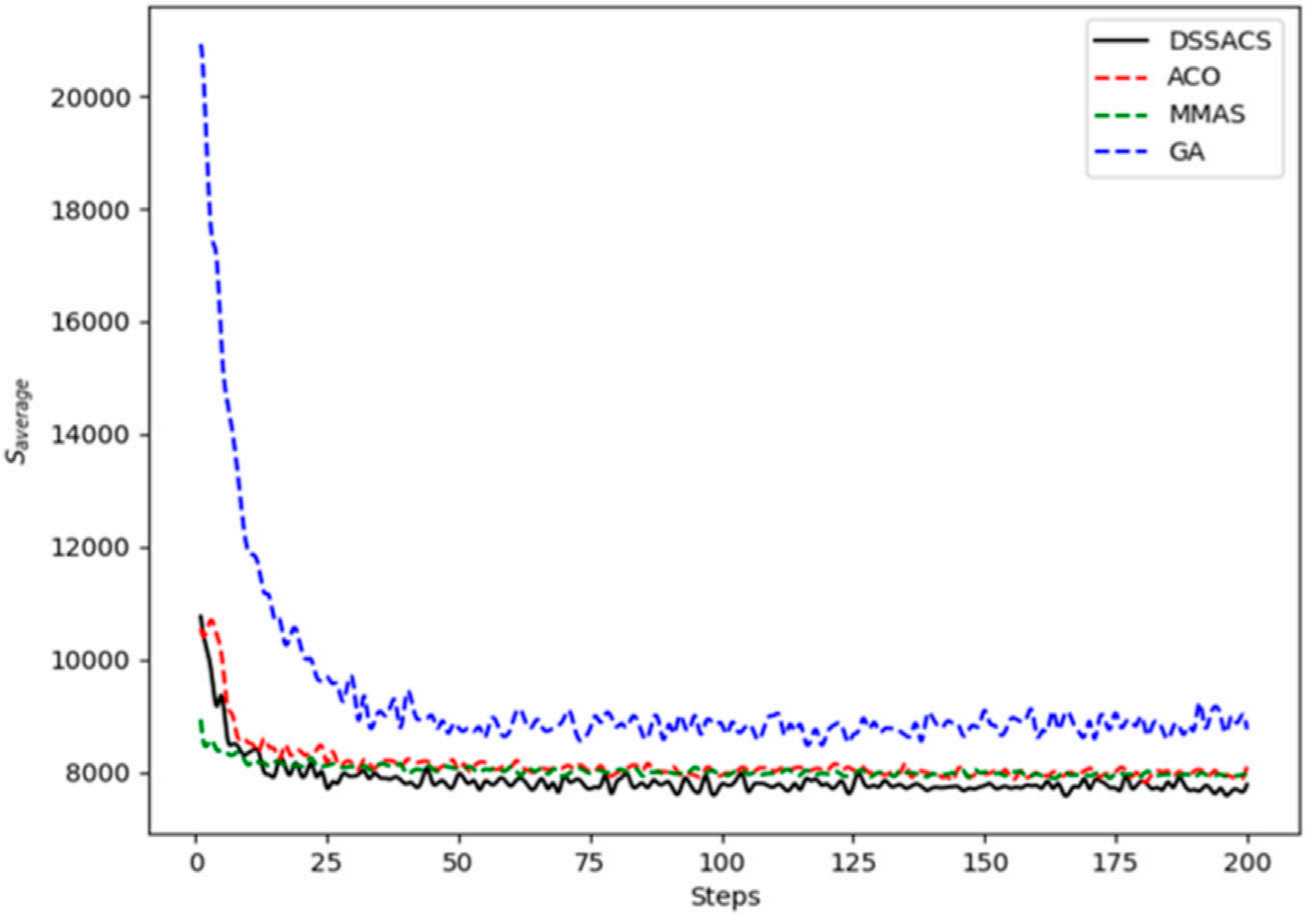
Berlin52.

**FIGURE 5:**
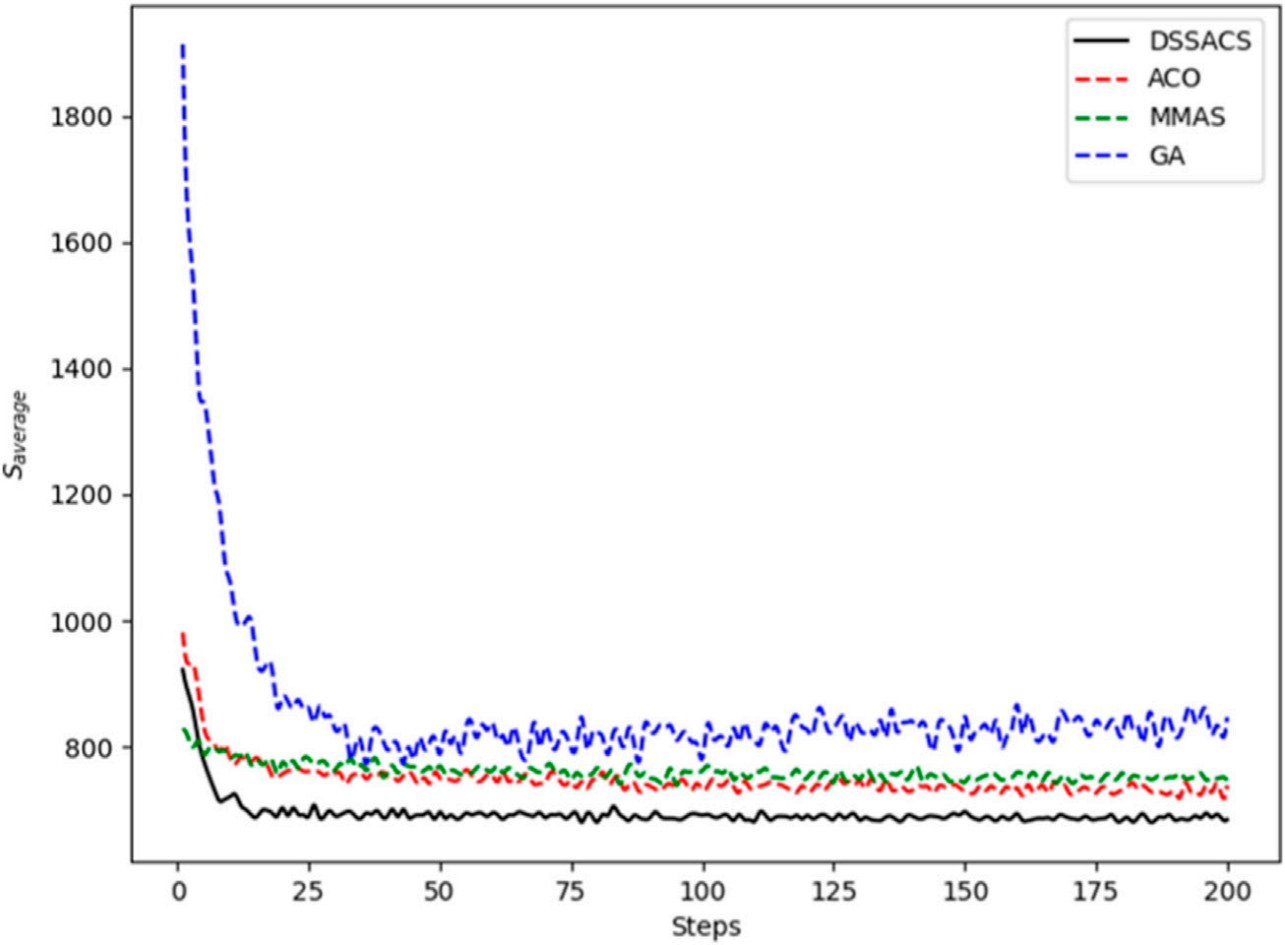
Dantzig42.

**FIGURE 6:**
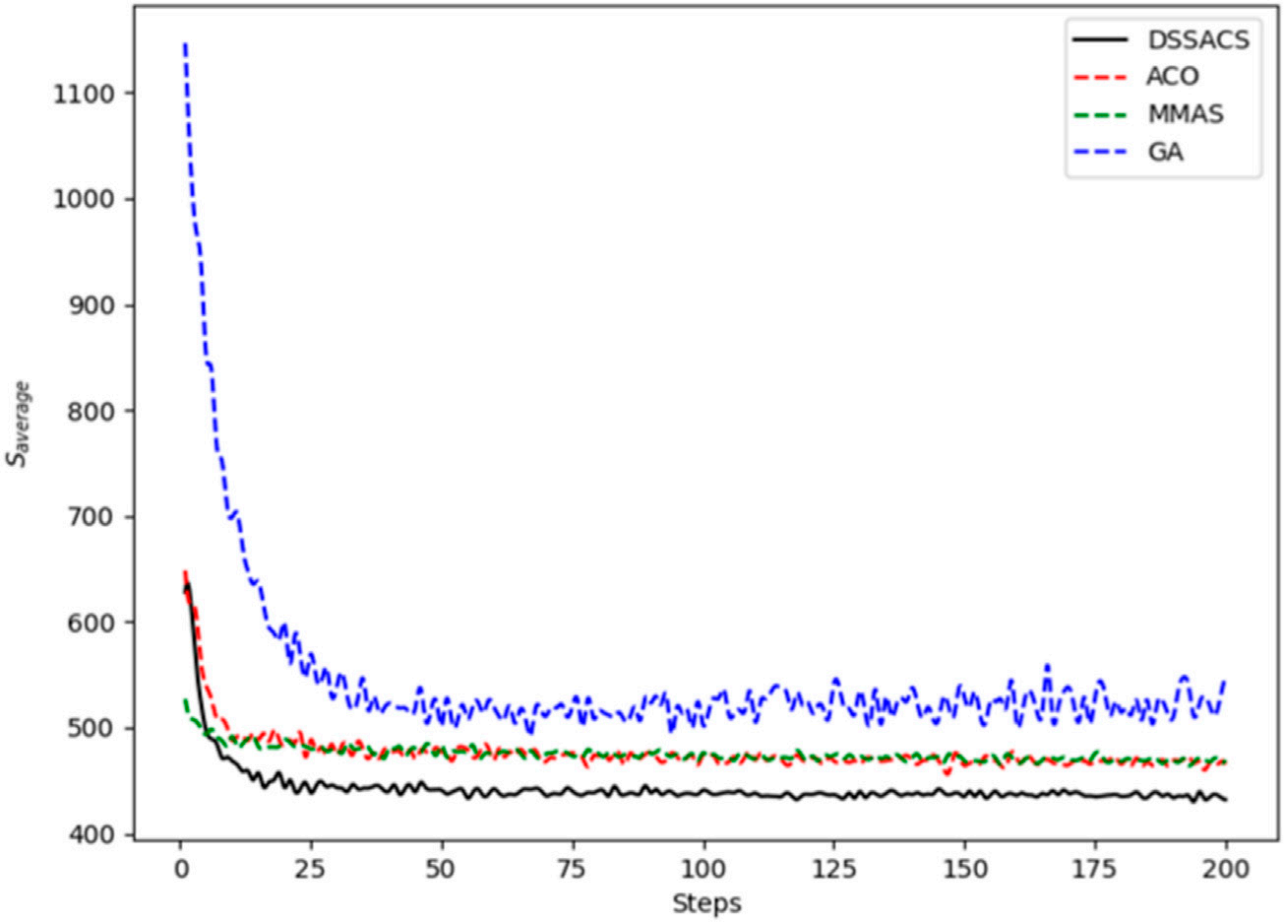
Eil51.

**FIGURE 7:**
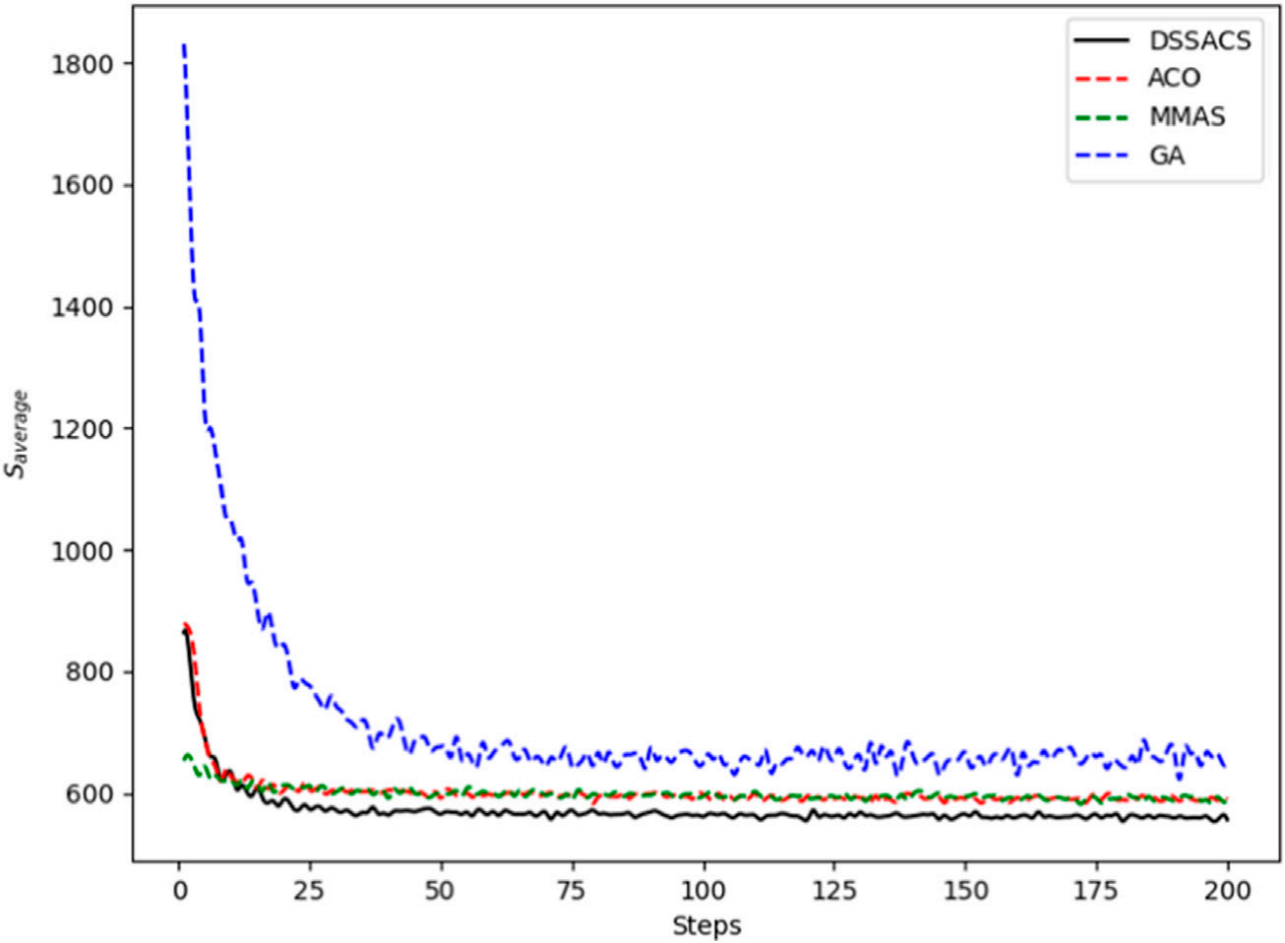
Eil76.

**FIGURE 8:**
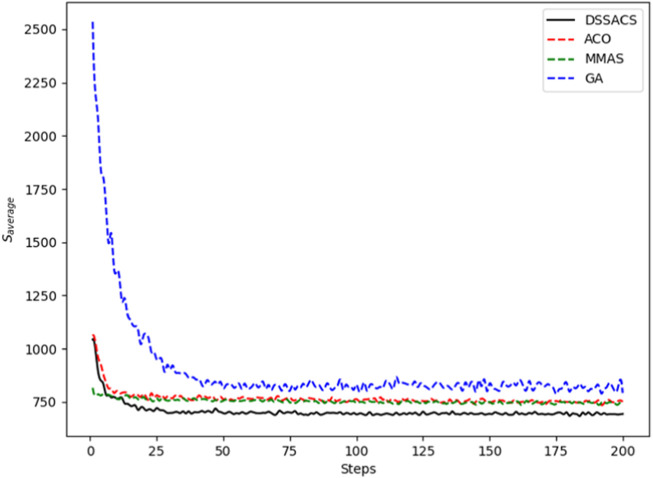
St70.

In Figures 3–8, we draw the operation diagram of DSSACS together with ACO, GA, and MMAS. These figures depict the change of $S_{average}$ as the maximum number of iterations of *Steps* increases. All data are averaged for ten times. We apply these algorithms to the TSP problems of Dantzig42, Eil51, Berlin52, Att48, St70, and Eil76.

The average variation curves of optimal solutions obtained by the four algorithms in the process of iteration can be seen. The decline of DSSACS is more obvious than ACO, GA, and MMAS, and the convergence rate is faster. In the early stage, the average value of DSSACS is lower than the other three functions, and with the increase in step size, the average value of DSSACS is also lower than ACO, GA, and MMAS. Compared with the other three algorithms, DSSACS has a faster convergence speed and higher solution quality, and a more excellent positive feedback mechanism. Compared with other ant colony algorithms, in the change of the maximum number of iterations, the optimal solution can always be 5%–6% lower than them, and compared with the genetic algorithm, this value can reach 15%–20%. DSSACS had a much higher search accuracy than other swarm intelligence algorithms. In our subsequent experiments, the algorithms corresponding to each TSP problem, such as DSSACS, GA, GA-PSO-ACO, etc., were averaged for 30 experiments to ensure the reliability of experimental data and their maximum iterations were set as 500 times each time. Table 2 summarizes the performance of each algorithm on the TSP problem. The optimal result represents the optimal distance obtained from TSPLIB, the best result represents the optimal value that an algorithm can obtain in 30 experiments, and the Average represents the average results over 30 times. Standard deviation means standard deviation of results, and AVR means average deviation rate.

**TABLE 2:** The comparison results of algorithms. (If the result of DSSACS is better than other algorithms, it will be marked in bold).

Test		DSSACS	GA	ACO	GA-PSO-ACO (Yelmewad et al., 2019)	Tabu search	PSO	FOGS-ACO (Gündüz et al., 2015)	DSMO (Saenphon et al., 2014)
ATT48 (33,523.7)	Best result	**33,523.7**	34,587	34,498	33,786	34,292	—	33,561.0	—
	Average	**33,783.54**	35,370	34,717	34,322	37,437	—	34,205.0	—
	Standard deviation	**219.27**	1,041.3	273.78	299.22	1,157.88	—	282.09	—
	AVR	0.78	2.30	0.63	1.59	9.17	—	1.92	—
EIL51 (426)	Best result	**426**	448.19	437.01	426	445.52	450.52	431.74	428.86
	Average	**432.33**	478.55	446.60	438.21	498.13	467.85	—	—
	Standard deviation	**2.94**	19.85	4.68	5.00	17.59	20.19	—	—
	AVR	**1.48**	6.77	2.19	2.87	11.81	3.85	—	—
BERLIN 52 (7,542)	Best result	**7,544.37**	8,289.58	7,647.56	7,544.37	7,973.60	8,157.39	7,544.37	7,544.37
	Average	7,631.52	8,400.17	7,696.30	7,591.88	8,315.91	8,288.44	—	—
	Standard deviation	142.58	128.75	74.70	53.13	174.99	136.60	—	—
	AVR	1.16	1.33	0.64	0.63	4.3	1.61	—	—
ST70 (677.11)	Best result	**677.11**	712.81	697.56	679.60	703.42	718.98	684.5	677.11
	Average	**689.94**	745.12	708.92	700.22	758.18	768.08	—	—
	Standard deviation	**6.85**	32.71	6.86	12.24	39.90	37.36	—	—
	AVR	1.89	4.53	1.63	3.03	7.78	6.83	—	—
EIL76 (538)	Best result	**544.86**	566.18	565.66	556.39	574.89	571.36	—	—
	Average	**553.88**	567.27	566.30	557.67	578.20	572.77	—	—
	Standard deviation	**6.04**	25.72	7.84	6.53	31.36	32.47	—	—
	AVR	1.66	0.37	0.11	0.23	0.58	0.24	—	—
RD100 (7,910)	Best result	**7,911.3**	8,138	8,258	—	8,171	8,295	—	—
	Average	**7,992.16**	8,418.56	8,453.18	—	8,442.67	8,604.86	—	—
	Standard deviation	**82.25**	217.63	109.01	—	254.02	234.83	—	—
	AVR	**1.0**2	3.45	2.36	—	3.32	3.74	—	—

The optimal solution of the DSSACS algorithm is superior to other algorithms is shown in Table2. The four indexes of DSSACS in Best result, Average, Standard deviation, and AVR show a great advantage. DSSACS can generally achieve the official optimal solution. For the TSP data sets, its average error is between 0.78% and 2.95% compared to the official optimal solution, while for other algorithms, the error is generally 2.03%–6.43%. DSSACS and GA-PSO-ACO achieved the same lowest optimal value in the case of EIL51. However, DSSACS is more stable than GA-PSO-ACO in the whole 30 calculation process, and it was easier to obtain the optimal solution. In EIL76, although DSSACS is more unstable than other algorithms, only DSSACS found the lowest optimal solution and the lowest average value in the same number of iterations, indicating that our algorithm can generally obtain the optimal solution.In general, DSSACS have faster convergence speed and higher solution quality.

We used the 2020 Shenzhen Cup Mathematical Modeling Competition^1^, as shown in Table 3. Point 0 is the base station in this system, where multiple wireless sensors collect data from the environment and send it to the data center of the base station. When the sensor’s power is lower than a certain threshold, the sensor cannot complete the regular sending and receiving tasks, and the WRSN network breaks down. The mobile charger needs to charge the sensor periodically to keep it from falling below the threshold for the WRSN to work correctly. The mobile charger starts from the data center and passes through each sensor at a fixed rate, charging the sensor at a fixed rate until it returns to the base station after charging all the sensors. Each sensor has a specific rate of energy consumption. The energy consumption of a mobile charger mainly has two aspects: one is the standard energy consumption caused by the charging sensor node; The other is the energy consumption of moving the charger on its way to charge the sensor. In order to reduce the energy consumption of mobile chargers on the road, it is necessary to plan the charging route of mobile chargers reasonably.

**TABLE 3:** Sensor coordinate dataset in WRSN.

No.	Longitude	Latitude	No.	Longitude	Latitude
0	120.7015202	36.37423	15	120.6960585	36.38247931
1	120.6987175	36.37457569	16	120.7005141	36.38276987
2	120.6997954	36.37591239	17	120.6998673	36.37079794
3	120.70691	36.37579616	18	120.6928965	36.37079794
4	120.7056165	36.37248342	19	120.6964897	36.36824059
5	120.7031731	36.37753964	20	120.6969209	36.37143727
6	120.6928965	36.37800457	21	120.7052571	36.36899618
7	120.6943337	36.37521499	22	120.7088504	36.37021674
8	120.6973521	36.37876006	23	120.7087066	36.36731063
9	120.6962022	36.37643544	24	120.7130185	36.36829872
10	120.7011609	36.37905063	25	120.6896626	36.36661314
11	120.6939026	36.37643544	26	120.6937588	36.36242812
12	120.6983582	36.38056159	27	120.6993643	36.38741865
13	120.7025263	36.38120084	28	120.7129466	36.37201847
14	120.6914592	36.38201441	29	120.7002266	36.38741865
					

First, the longitude and latitude of each sensor and base station have been informed in the link. First, we assume that the longitude and precision of sensor A are $lng_{A}、lat_{A}$ ,  B is similar to A . We set the radius of the Earth as R=6371Km , π=3.141592653589793 . According to the calculation formula of radian and sphere distance:

$$d_{AB}=2R\cdot \sin^{-1}\sqrt{\sin{\left(\frac{\varphi_{A}-\varphi_{B}}{2}\right)}^{2}+\cos\varphi_{A}\cos\varphi_{B}\sin{\left(\frac{\mu_{A}-\mu_{B}}{2}\right)}^{2}}\;$$ (3.1)

$$\varphi_{A\left(B\right)}=lat_{A\left(B\right)}\cdot \frac{\pi }{180}\;$$ (3.2)

$$\mu_{A\left(B\right)}=lng_{A\left(B\right)}\cdot \frac{\pi }{180}$$ (3.3)

First, we give a few measures and their definitions.

1) steps refer to the iterations of the algorithm. The higher the number of iterations of *steps*, the higher the algorithm’s accuracy.
2) $S_{average}$ uses an algorithm to compute the exact MTSP  n  times and find their average. The calculation formula of $S_{average}$ is:

$$S_{average}=\overset{n}{\underset{i}{\sum }}\frac{best_{i}}{n}$$ (3.4)

3) $S_{total}$ refers to the sum of the paths chosen by all salps populations after each iteration. Suppose that there are *n* iterations in total and *s* salps in a population, then there is the formula:

$$S_{total}=\overset{s}{\underset{i}{\sum }}ant_{t=t_{0}}^{i}$$ (3.5)

$t_{0}$ represents the current iteration number, and $\;ant_{t=t_{0}}^{i}$ represents the total path length selected by ant *i* at $\;t_{0}$ iteration. In general, the value of $S_{total}$ should be smaller when the positive feedback mechanism of the algorithm is more robust. We set the MCV data as follows:

1) The moving speed of the MCV is v=5m/s

2) The charging rate of the MCV is *R* = 400 mA/h
3) The lowest battery capacity of the sensor *w* = 7.3 mA
4) The number of MCVS, *m* = 4

**FIGURE 9:**
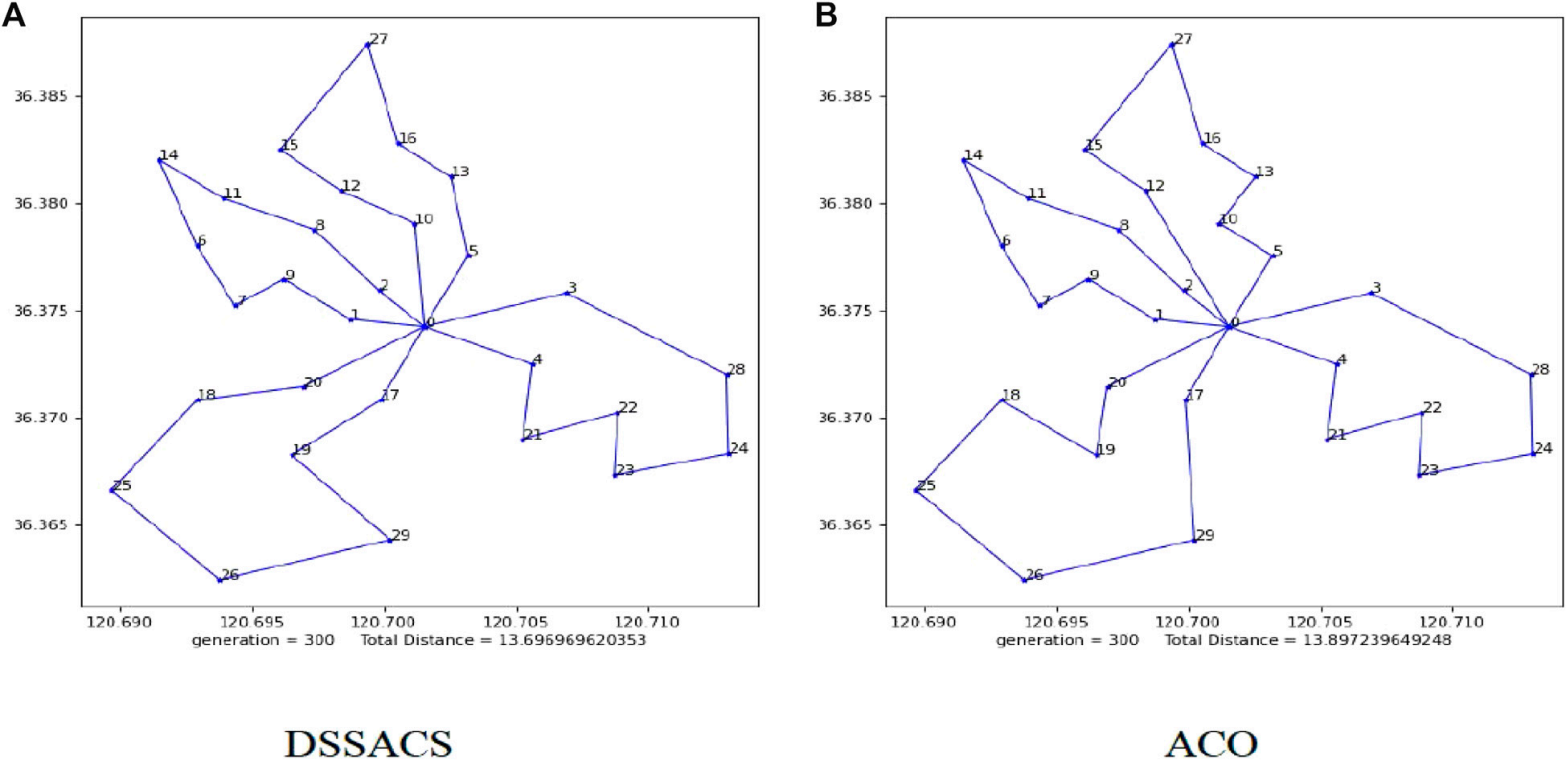
Paths of DSSACS **(A)** and ACO **(B).**

First, the optimal solution is 13.697 km under the maximum number of 300 iterations shown in Figure 9. The DSSACS has a high convergence speed compared with ACO, while DSSACS can be completed in the early iteration. Moreover, the ACO is not as good as DSSACS in some image details. It is longer than SSDACD in overall path length, leading to increased wireless sensor battery capacity and high cost.

**FIGURE 10:**
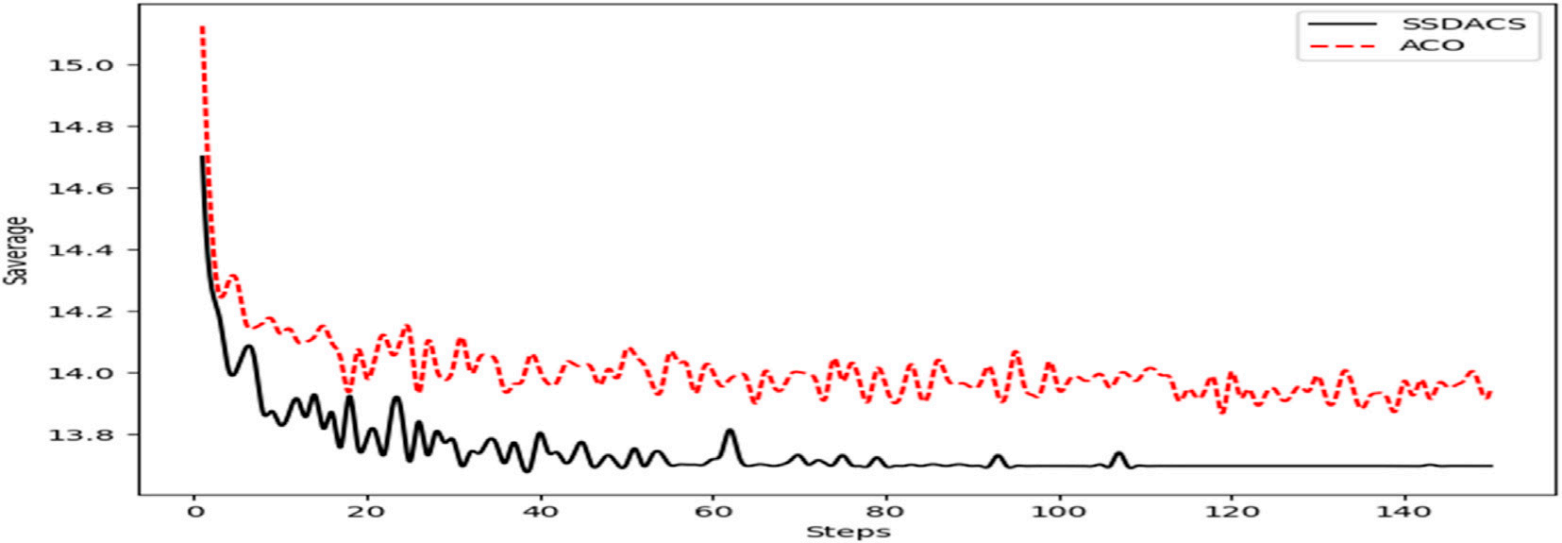
The comparison of the convergence process of $S_{average}$ among DSSACS and ACO.

In Figure 10, the change of $S_{average}$ of ACO and DSSACS with the increase of iteration *steps* is plotted. It can be seen that at the beginning of the iteration, DSSACS and ACO have a significant difference. Compared with ACO, DSSACS has a shorter average path length and a faster convergence speed. In the subsequent iterations, DSSACS showed more significant advantages than ACO. After 110 generations, the images of DSSACS almost become a straight line, and the optimal solution can be reached almost every time, while ACO is still in the overall decline stage. Overall, the convergence speed of DSSACS is better than that of ACO in all iterations.

**FIGURE 11:**
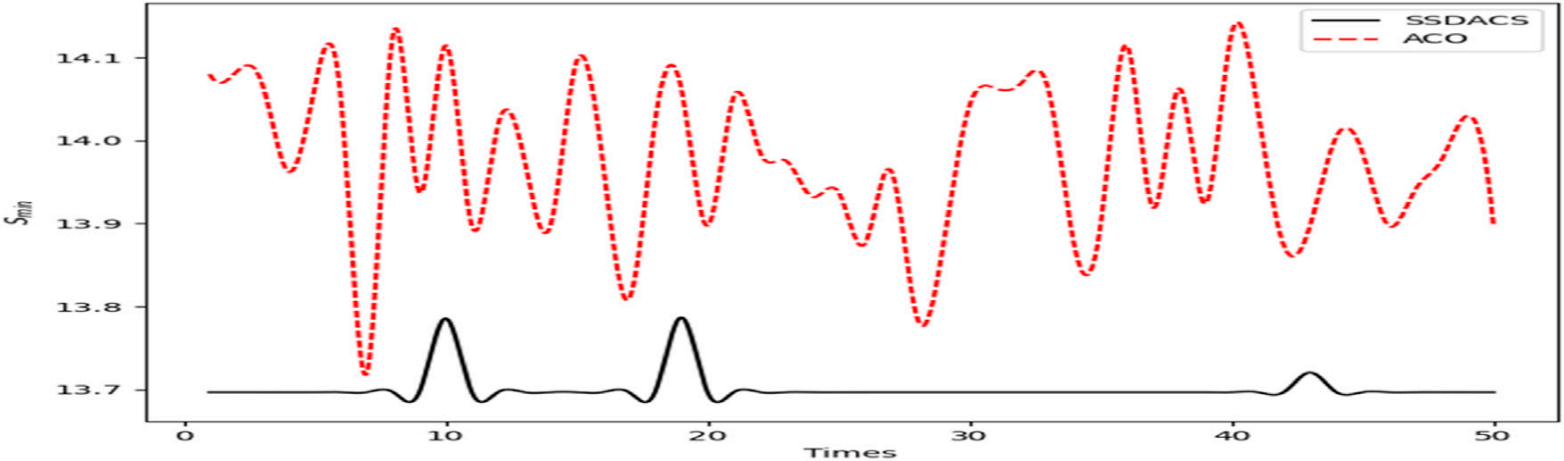
The comparison of $S_{min}$ 50 times between DSSACS and ACO.

In Figure 11, we plotted the change of $S_{min}$ with the increase of *Times*. We set a fixed number of iterations *steps* = 100, and conducted 50 experiments on both algorithms. The Figure shows that in the case of fewer iterations, DSSACS has almost reached the optimal value, and the DSSACS algorithm is very stable within 100 iterations. Compared with DSSACS, the ACO algorithm is more unstable in the early stage and fails to reach the optimal solution. For example, most ACO’s are above 13900m, while DSSACS have never reached this value.

**FIGURE 12:**
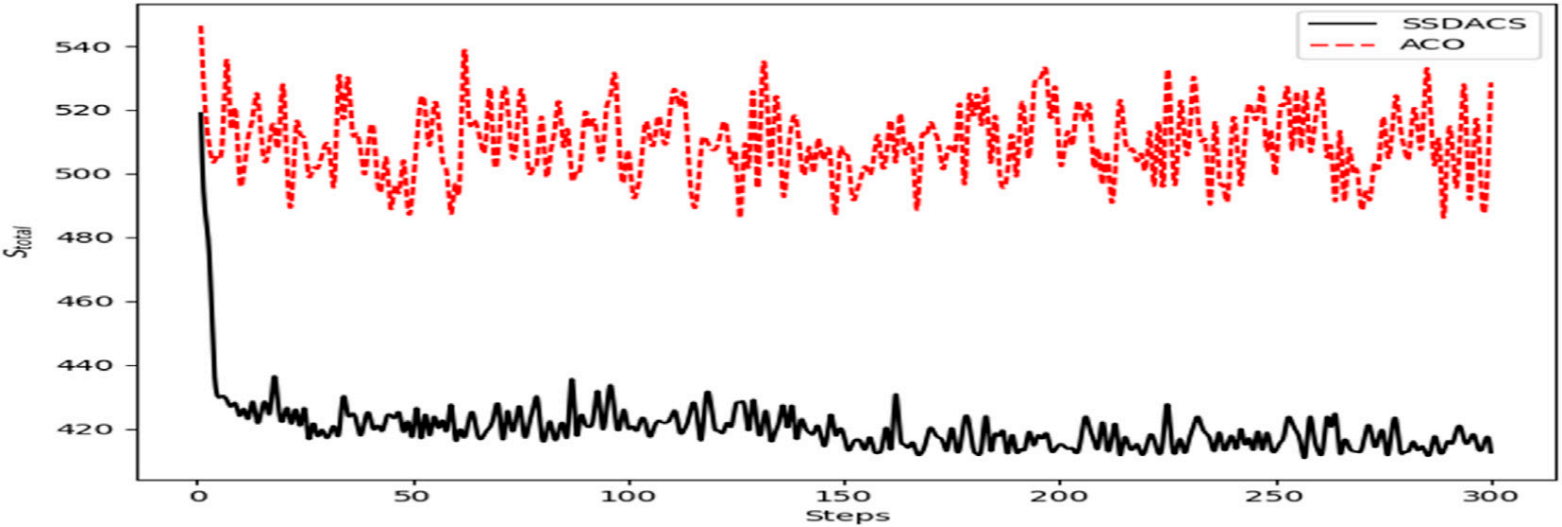
The comparison of the reflection of positive feedback mechanism ( $S_{total}$ ) between DSSACS and ACO.

Finally, we compared the change of $S_{total}$ as. The iteration *steps* increased, as shown in Figure 12. At the beginning of the iteration, the slope of DSSACS is significantly greater than ACO, indicating that the former has a faster convergence rate. Moreover, from the beginning of the iteration, the path selected by salps arithmetic in DSSACS was better than that chosen by ants in ACO, indicating that the positive feedback mechanism of DSSACS was more robust, which enabled the algorithm to maintain a better path selection in the whole iteration process.

According to Eqs 2.1.–.2.7, the maximum battery capacity obtained by using DSSACS $\underset{1\leq i\leq n}{\max}W_{i}=9.13\text{mA}$ is the minimum capacity in all circuits. Using DSSACS can not only achieve the minimum path cost but also improve the charging efficiency. At this point, the four paths of MCV are shown in fellow, and the lengths of the four distances are 3.35, 3.49, 4.01, and 2.85 km respectively. The corresponding optimal charging paths are as follows:

: $\;BS\to S_{1}\to S_{9}\to S_{7}\to S_{6}\to S_{14}\to S_{11}\to S_{8}\to S_{2}\to BS$

: $\;BS\to S_{10}\to S_{12}\to S_{15}\to S_{27}\to S_{16}\to S_{13}\to S_{5}\to BS$

: $\;BS\to S_{3}\to S_{28}\to S_{24}\to S_{23}\to S_{22}\to S_{21}\to S_{4}\to BS$

: $\;BS\to S_{20}\to S_{18}\to S_{25}\to S_{26}\to S_{29}\to S_{19}\to S_{17}\to BS$

## 5 Summary and outlook

This paper proposes a discrete optimization strategy for Salps based on the ant colony system. Our optimized DSSACS algorithm is applied to solve the application of the TSP problem and MTSP problem. We added the advantage of population initialization from the ant colony system to the leader of the salp colony system to solve the problem of the disorder and confusion in the initialization of the salp swarm algorithm. Thus, significantly improving the correlation and purpose between the population and optimizing the follower strategy of the salp colony to improve the convergence speed of the algorithm. We first apply DSSACS to the MCV path planning problem of WRSN networks. The charging problem of WRSN networks can be regarded as an MTSP problem. DSSACS can improve the algorithm’s calculation speed, save time and economic cost of the WRSN network by planning the path. DSSACS surpasses the ACO algorithm in terms of stability, convergence speed, and accuracy in terms of overall performance. Then we compare DSSACS with other metaheuristic algorithms on the TSP problem. The optimal and average solutions obtained by DSSACS are superior to other algorithms, and the SSACS algorithm is almost the best in convergence speed, robustness, and positive feedback mechanism. Our experiments show that DSSACS is feasible and effective in solving NP-hard problems. Although the algorithm proposed in this paper has a significant improvement compared to the original algorithm, it is only used in the field of wireless charging in this paper. It is believed that through the potential of DSSACS, it can break through the barriers in other fields and play a role in other fields in the future.

## Data Availability

The raw data supporting the conclusions of this article will be made available by the authors, without undue reservation.

## Footnotes

1
http://www.m2ct.org/viewpage.jsp?editId=12&uri=0D00233&gobackUrl=modulalist.jsp&pageType=smxly&menuType=flowUp1, 2020.
